# Machine learning of immune-related laboratory indicators enables discrimination between pulmonary and extrapulmonary tuberculosis

**DOI:** 10.3389/fmed.2025.1712073

**Published:** 2025-12-16

**Authors:** Shuanglin Gu, Yichen Guo, Han Jiang, Chen Yang, Tengyue Li, Xiuchang Ma, Wenxian Qian, Zihao Liang, Zheng Hu, Apeng Chen, Yi Zeng, Changhua Yi

**Affiliations:** 1 Department of Clinical Research Center, The Second Hospital of Nanjing, Affiliated to Nanjing University of Chinese Medicine, Nanjing, China; 2 Department of Tuberculosis, The School of Public Health of Nanjing Medical University, The Second Hospital of Nanjing, Nanjing, China; 3 Department of Bioinformatics Science and Technology, Harbin Medical University, Harbin, China; 4 Nanjing Key Laboratory of Pediatrics, Children’s Hospital of Nanjing Medical University, Nanjing, China; 5 Department of Respiratory, Children’s Hospital of Nanjing Medical University, Nanjing, China; 6 Department of Tuberculosis, The Second Hospital of Nanjing, Affiliated to Nanjing University of Chinese Medicine, Nanjing, China

**Keywords:** diagnostic prediction, extrapulmonary tuberculosis, immune-related laboratory indicators, machine learning, model explanation

## Abstract

**Introduction:**

As a globally significant infectious disease, the tuberculosis bacterium can not only cause pulmonary tuberculosis through respiratory tract infection but also spread from pulmonary lesions to organs throughout the body via the blood or lymphatic system, resulting in extrapulmonary tuberculosis (EPTB). However, achieving early and accurate diagnosis of EPTB remains a major clinical challenge.

**Methods:**

In this study, 1,580 patients were identified as samples for analysis. Through the process of decollinearity and characterization, seven routine blood markers: Basophil (BASO), Hemoglobin (HB), Mean erythrocyte hemoglobin concentration (MCHC), mean corpuscular volume (MCV), mean platelet volume (MPV), Red Blood Cell (RBC), Lymph and red blood cell distribution width coefficient of variation (RDW-CV), one cytokine: interleukin-6 (IL-6), and three lymphocyte-related indices: CD4+ T cell, CD4+/CD8+ T cells, CD8+ T cells were included in the subsequent model construction. Then nine machine learning (ML) algorithms were used to construct predictive models.

**Results:**

The most outstanding one is the K-Nearest Neighbors (KNN) model, which has an AUC value as high as 0.846, with sensitivity and specificity of 0.769 and 0.786, respectively, shows strong prediction effectiveness. Among them, CD4+ T cells, HB, and MPV ranked the top three in terms of importance.

**Discussion:**

In conclusion, we have developed an interpretable ML model to identify EPTB based on data from routine laboratory tests, which provides some assistance in early diagnosis.

## Introduction

For the past 25 years, tuberculosis (TB) has been considered as a global public health emergency. Each year, there are over 10 million new TB cases, and it ranks among the top 10 causes of death globally. According to the World Health Organization’s Global Tuberculosis Report 2024, approximately 10.7 million new TB cases occurred globally in 2024, including 5.8 million in males, 3.7 million in females, and 1.2 million in children. Around 8.3 million cases were newly diagnosed and enrolled in treatment, marking a historic high and covering about 78% of the estimated incidence (1).

The development of tuberculosis is closely related to immune response. *Mycobacterium tuberculosis* divergens is the predominant causative agent of tuberculosis. Transmission of this bacterium occurs almost solely via droplet infection (2). Once inhaled, bacterial growth predominantly occurs in the upper regions of the well-ventilated lung, specifically within alveolar macrophages. As the infection persists over time, the immune response is triggered, and T cells are generated. This leads to a slowdown in bacterial growth; however, at this stage, the bacteria can still survive, despite not causing any clinical symptoms (3). Extrapulmonary tuberculosis is associated with underlying immunodeficiency. For example, HIV-infected individuals are at increased risk of developing extrapulmonary TB, and this risk increases as CD4+ T-lymphocyte counts decline. There is also an increased incidence of extrapulmonary TB in young children, particularly tuberculous meningitis, which may be due to immaturity of the immune system (4, 5). The human immunodeficiency virus (HIV) can heighten the risk of active tuberculosis up to 26-fold, significantly complicating both the diagnosis and treatment processes (6). This may be because HIV increases the risk of active TB and its extrapulmonary spread through multiple mechanisms. It inhibits macrophage apoptosis, depletes CD4+ T lymphocytes, which impacts the functional structure of granulomas, and elicits other intricate immune responses. As a result, the body struggles to restrict the growth and spread of TB effectively (7).

When alveolar macrophages phagocytose *Mycobacterium tuberculosis*, the bacterium proliferates in the interstitial space between macrophages and alveoli. Subsequently, new monocytes migrate from the bloodstream to the site of the lesion. Macrophage-mediated transport of mycobacteria then occurs towards neighboring lymph nodes. From the lymph nodes, the bacilli can disseminate to the subclavian vein and subsequently throughout the body via the hematogenous pathway. Employing the lymphohematogenous route, new foci of infection are established until an immune response is initiated. Consequently, various organs may become involved. Extrapulmonary manifestations may or may not be associated with pulmonary infection. Extrapulmonary tuberculosis (EPTB) can affect any organ or tissue, except for hair, nails, and teeth (8). One in five cases of TB presents with EPTB, which is most common in the lymph nodes (50%), pleura (18%), genitourinary system (13%), bones and joints (6%), gastrointestinal system (6%), central nervous system (CNS) (3%), and spine (3%) (9).

Globally, the proportion of extrapulmonary tuberculosis cases reported by different countries varies from 15 to 25%. This proportion is notably higher in countries with a high prevalence of HIV infection. Besides being relatively more common among HIV-infected individuals, extrapulmonary involvement occurs more frequently in children compared to adults (10). The clinical manifestations of EPTB vary significantly depending on the site of the infection and the disease’s aggressiveness. Moreover, its features and symptoms are nonspecific, making its accurate diagnosis a persistent challenge. The most fundamental diagnostic approach is microscopy for detecting *M. tuberculosis*. However, this method is limited in sensitivity and exhibits poor specificity in cases of oligomycobacterial tuberculosis, such as childhood tuberculosis, EPTB, or tuberculosis co-infected with HIV (11).

Machine learning algorithms, as artificial intelligence techniques, function by choosing the optimal model from a set of alternatives to fit a collection of observations. Through training, machine learning (ML) automatically discerns patterns and features within data and generates predictive and decision-making models. These models can then be applied to predict outcomes and classify new data (12). Over the past decade, the application of machine learning in medicine has experienced exponential growth and has been implemented across a wide range of medical fields (13). Recent advances in artificial intelligence (AI), particularly ML, have revolutionized TB diagnostics by leveraging high-dimensional biological data to overcome the limitations of traditional methods. Between 2022 and 2025, pivotal studies have demonstrated ML’s potential in TB subtyping and EPTB detection. Luo et al. established a machine learning-based diagnostic model for distinguishing active tuberculosis (ATB) from latent tuberculosis infection (LTBI) (14). Shao et al. developed a multimodal integration (MMI) system that combines clinical, imaging, and laboratory testing data to diagnose various pulmonary infections and pathogens. The system achieved AUC values of 0.910 (95% CI: 0.904–0.916) in internal test datasets and 0.887 (95% CI: 0.867–0.909) in external test datasets, which are comparable to the performance of experienced physicians (15).

Building upon these developments, our study aims to development of interpretable ML models that leverage routinely available laboratory parameters rather than specialized omics or imaging data. This research therefore seeks to bridge the gap between basic TB immunology and advanced ML applications, with the ultimate goal of providing a practical diagnostic tool that can be implemented in diverse healthcare settings to improve EPTB diagnosis and patient outcomes.

## Materials and methods

### Study population

The study cohort for this study was derived from the patient population of the tuberculosis department of Nanjing Second Hospital (public health medical center).

This study retrospectively enrolled tuberculosis patients who were hospitalized from 2021 to 2023. Patients were eligible for inclusion if they met the clinical diagnostic criteria for TB and presented at least one of the following confirmatory evidence: (1) positive acid-fast bacilli (AFB) smear, (2) positive mycobacterial culture, or (3) positive molecular biology tests [Xpert MTB/RIF or other nucleic acid amplification tests (NAAT)]; alternatively, patients with pathological biopsy demonstrating tuberculous granulomas and positive AFB staining, along with characteristic tuberculous lesions on imaging, were also included. For patients with EPTB, in addition to the above criteria, a biopsy of the affected lesion (pathology + acid-fast staining) or positive pathogen detection in the body fluids of the involved organ was required, accompanied by local symptoms and abnormal imaging findings in the corresponding organ. Patients were excluded if they: (1) had not undergone blood routine tests, cytokine tests, and cellular subset tests simultaneously, (2) had any missing values in the inclusion characteristics, or (3) were infected with HIV (regardless of test result positivity or clinical suspicion of HIV infection). This study was conducted in strict compliance with the Code of Medical Ethics and was approved by the Ethics Committee of Nanjing Second Hospital.

### Data collection and processing

Based on electronic medical record data from the hospital information system (HIS), feature screening and prediction model construction were systematically carried out by integrating patients’ demographic characteristics, laboratory biochemical indexes, and data from their first admission test.

A total of 48 characteristics were obtained, which can be categorized into three main groups: hematological parameters, cytokine levels, and lymphocyte subpopulation metrics. Hematological Parameters: including the Basophil (BASO), Basophil percentage (BASO%), absolute eosinophil count (EOS), eosinophil percentage (EOS%), packed Cell Volume (PCV), hemoglobin (HB), absolute lymphocyte count (Lymph), lymphocyte percentage (Lymph%), mean hemoglobin content (MCH), mean hemoglobin concentration (MCHC), mean corpuscular volume (MCV), monocytes (MONO), monocyte percentage (MONO%), mean platelet volume (MPV), absolute neutrophil count (ANC), glucocorticoid receptor percentage (GR%), procalcitonin (PCT), platelet distribution width (PDW), prolactin (PRL), red blood cell(RBC), coefficient variation of red blood cell distribution width (RDW-CV), and white blood cell (WBC). Cytokine Levels: Measured cytokines encompassed interleukin-4 (IL-4), interleukin-6 (IL-6), interleukin-10 (IL-10), tumor necrosis factor-alpha (TNF-*α*), interferon-gamma (IFN-*γ*), interleukin-17a (IL-17a), interleukin-1β (IL-1β), interleukin-5 (IL-5), interferon-alpha (IFN-α), and interleukin-8 (IL-8). Lymphocyte Subpopulation Metrics: This group included the percentage of CD3+ T cells (CD3+ %), absolute count of CD3+ T cells, CD8+/CD45+, CD8+ T cells, CD4+/CD45 +cells, CD4+ T cells, CD4+ CD8+/CD45+ cells, CD4+CD8+ T cells, percentage of CD16/56+ natural killer (NK) cells, CD16/56+ NK cells, CD19+ B cells, CD45+ cells, percentage of CD19+ B cells, CD45+ lymphocytes, and the Th/Ts (CD4+/CD8+).

At the stage of statistical analysis, the median (with interquartile spacing) was used to describe continuous variables with skewed distributions statistically. We used the Mann–Whitney U test (two-tailed test) to compare the distribution differences between the two groups and calculated the original–values. To control the risk of false–positive results caused by multiple comparisons, we employed the False Discovery Rate to correct the original–values. This correction process was implemented through the *p*.adjust function (method = “fdr”), and we set a corrected as the strict threshold for statistically significant differences. The initial 48 features were refined in two steps to improve data quality and reduce multicollinearity. First, constant features (single unique value) were excluded for lack of discriminative information. Second, highly collinear features (pairwise Pearson correlation >0.8) were removed using the findCorrelation function (caret package, version 7.0.1). Subsequently, LASSO regularization (glmnet package, version 4.1.10) was applied for predictive feature selection. A 5-fold cross-validation (CV) was used to determine the optimal regularization parameter (*λ*), and only features with non-zero coefficients at λ.min (minimum CV error) were retained. These features are considered to have significant associations with the disease states studied.

To avoid data leakage and overfitting, a fixed random seed (456) was set for reproducibility. To prevent data leakage and overfitting, a fixed random seed 456 was set for reproducibility, the original dataset was split into a 70% original training set and a 30% independent test set before any oversampling, preprocessing, or hyperparameter tuning. Only the original training set was oversampled using the upSample function from the caret package to create a balanced training set with equal numbers of negative and positive cases. The test set remained untouched, and numerical features were standardized (centered to mean = 0, scaled to SD = 1) using the preprocess function, applied to the training set and propagated to the test set.

### Building diagnostic models and model explanation

In this study, nine classical machine learning models were trained for predicting primary and secondary outcomes, namely: Logistic Regression, Random Forest (RF), Linear Discriminant Analysis (LDA), Support Vector Machine (SVM), Decision Tree (DT), Gradient Boosting Machine (GBM), Naive Bayes, K-Nearest Neighbors (KNN), and eXtreme Gradient Boosting (XGBoost) (16, 17). The objective was to accurately classify and predict patients with pulmonary tuberculosis versus those with non-pulmonary tuberculosis. Model performance evaluation employed a hierarchical system. The area under the curve (AUC) served as the primary evaluation metric, complemented by secondary indicators such as accuracy, sensitivity, specificity, positive predictive value (PPV), and negative predictive value (NPV) for a comprehensive assessment. Each model used 5-fold stratified cross-validation to optimize hyperparameters and assess generalization, with model optimization performed exclusively on the training set and the independent test set reserved for final evaluation. We have analyzed the feature importance using SHAP (SHapley Additive exPlanations) analysis, which provides a more robust and interpretable ranking of feature contributions based on model predictions. This was implemented using the fastshap package (version 0.1.1) in R, which efficiently calculates SHAP values designed for interpreting the predictive results of machine learning models and is particularly suited for fast processing of tree models. To address potential overfitting, we calculated AUC confidence intervals (95% CI) for all models via bootstrapping (1,000 replicates, BCA method as primary, normal approximation as fallback). We then employed decision curve analysis (DCA) to evaluate the clinical utility of each predictive model on the test set. This involved quantifying the net benefit (the difference between the benefits of correctly identifying positive cases and the harms of incorrectly labeling negative cases) across different threshold probabilities, and comparing the clinical benefits of each model with the “treat all” and “treat none” strategies. Prediction probabilities were preprocessed according to model output characteristics SVM probabilities were smoothed using moving average and linearly calibrated, DT probabilities were extracted as the second-class probability or defaulted to 0.5, XGBoost probabilities were taken from the optimal iteration, and other models directly used predict (type = “prob”), and the net benefit for each model and reference strategies (all classified as positive/all classified as negative) was calculated across threshold probabilities ranging from 0.0 to 1.0 with a step size of 0.005. DCA curves were plotted using ggplot2 package.

## Results

### Clinical characteristics of patients

**Figure 1:**
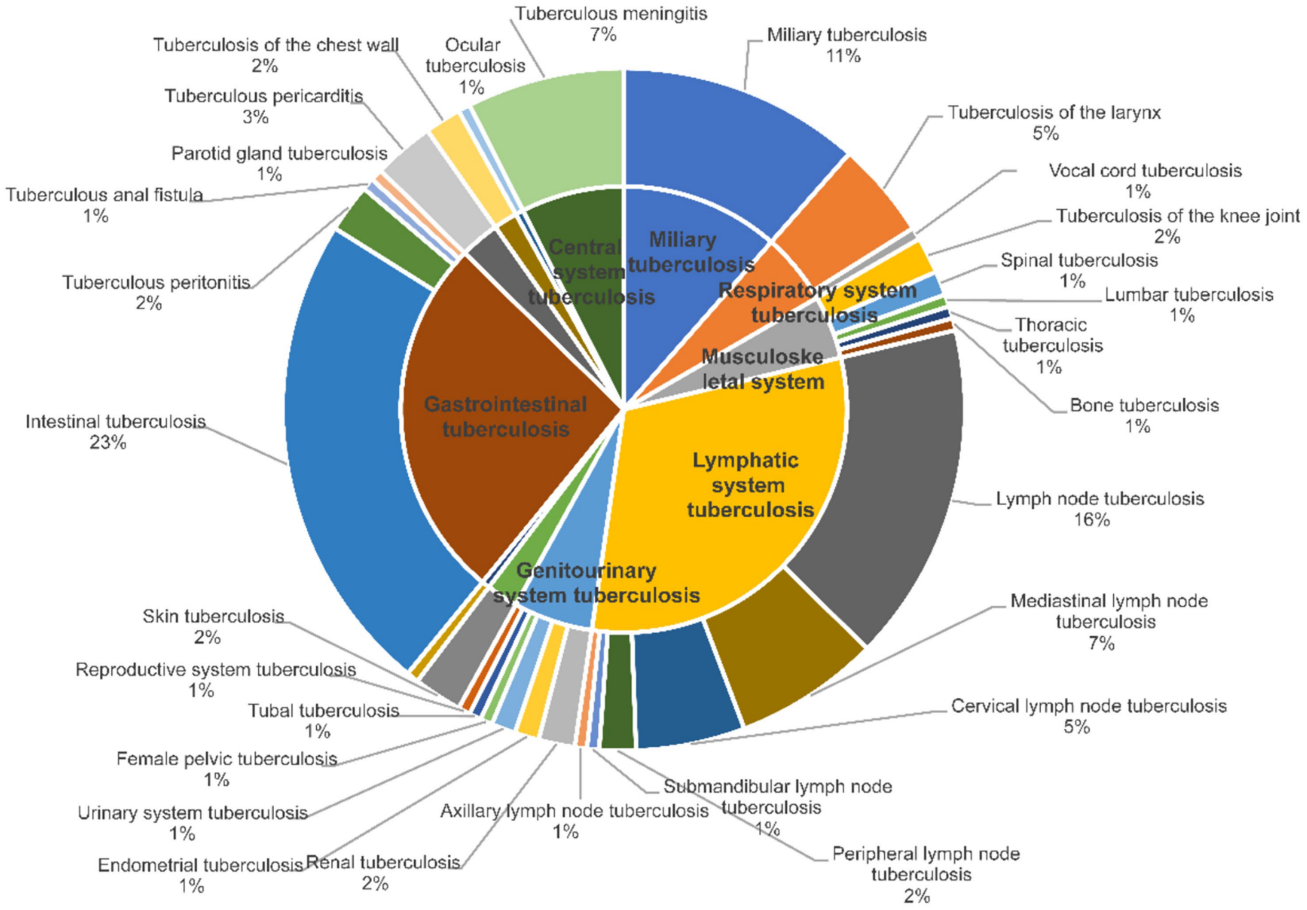
Percentage of EPTB by subtype. The inner circle of the pie chart shows the classification of nodules at the system level, and the outer circle shows specific nodule types.

This study enrolled a total of 5,056 tuberculosis patients. Based on the selected 46 predictors, the researchers finally selected 1,580 patients as the sample for analysis. Among these 1,580 patients, 174 (11.01%) were diagnosed with extrapulmonary tuberculosis (EPTB). Regarding gender differences, the number of female tuberculosis patients was 619, accounting for 39.15% of the total. Specifically, 545 of them had pulmonary tuberculosis (PTB), representing 38.76% of female patients, while 74 suffered from EPTB. There were 961 male tuberculosis patients, accounting for 60.85% of the cohort. Of these male patients, 861 had PTB and 100 had EPTB. Overall, the number of male tuberculosis patients slightly exceeded that of females, and the prevalence of EPTB was markedly lower than that of PTB. When categorized by age, 507 patients (32.09%) were under 40 years old. Among them, 446 had PTB and 61 had EPTB. There were 507 patients aged between 40 and 60 years, with 453 having PTB and 54 having EPTB. For patients over 60 years old (*n* = 566), 507 had PTB and 59 had EPTB. Our findings suggest that the incidence of tuberculosis infection declines to some extent among patients aged over 60 (Table 1). When classifying the types of tuberculosis in patients with EPTB at the systemic level, it was found that the proportion of patients with lymphatic system tuberculosis was the highest, accounting for 31.6%. This was followed by digestive system tuberculosis, which accounted for 25.9% of the patients. Additionally, a certain percentage of patients were infected at more than one site (Figure 1).

**Table 1:** Distribution of tuberculosis patients.

		Overall	PTB	EPTB
Number (%)		1,580	1,406 (88.99)	174 (11.01)
Sex (%)	F	619 (39.15)	545	74
	M	961 (60.85)	861	100
Age (%)	<40	507 (32.09)	446	61
	40–60	507 (32.09)	453	54
	>60	566 (35.82)	507	59

Using *p* < 0.05 as the criterion for determining statistically significant differences. Out of 48 markers, around 19 showed significant differences (13 hematological, 1 cytokine, 5 lymphocyte subsets). A total of 13 hematological indicators showed significant differences between groups after adjustment, mainly involving leukocyte subsets (such as lymphocytes and monocytes), red blood cell-related parameters (such as PCV, MCV, MCH, RDW-CV), and platelet-related parameters (such as MPV, PDW). This suggests that there are certain differences in blood composition between TB and EPTB patients, especially in lymphocyte and red blood cell parameters. Among the 17 cytokines, only IL-6 showed a significant difference between groups after adjustment (*p* = 0.0091), suggesting that IL-6 may play a differential role in the immune response between TB and EPTB. The remaining cytokines did not show significant differences between the two groups, and most of the adjusted *p*-values were high, indicating that the expression of these inflammatory factors is relatively similar between the two groups. Five lymphocyte subset indicators showed significant intergroup differences after adjustment, mainly involving the total T cell count (CD3+ T cells), helper T cells (CD4+ T cells), suppressor T cells (CD8+ T cells), total lymphocyte count (CD45+ T cells), and absolute number of B cells (CD19+ B cells). These differences suggest that there are certain distinctions between TB and EPTB patients in terms of cellular numbers in lymphocyte immune responses, particularly notable changes in the absolute numbers of T cell subsets (Tables 2–4).

**Table 2:** Comparison of hematological parameters between PTB and EPTB patients.

Variable	Overall (*n* = 1,580)	TB (*n* = 1,406)	EPTB (*n* = 174)	adj.*p*-value (FDR)
BASO, M (Q₁, Q₃)	0.03 (0.02–0.04)	0.03 (0.02–0.04)	0.02 (0.01–0.03)	0.0037
BASO%, M (Q₁, Q₃)	0.40 (0.25–0.55)	0.40 (0.25–0.55)	0.40 (0.20–0.60)	0.0068
EOS, M (Q₁, Q₃)	0.11 (0.04–0.17)	0.11 (0.04–0.18)	0.10 (0.04–0.16)	0.0385
EOS%, M (Q₁, Q₃)	1.90 (0.79–3.01)	1.90 (0.80–3.00)	1.70 (0.50–2.90)	0.0685
PCV, M (Q₁, Q₃)	37.50 (34.05–40.95)	37.70 (34.30–41.10)	36.60 (32.86–40.34)	0.0049
HB, M (Q₁, Q₃)	47.65 (3.90–91.40)	46.05 (2.30–89.80)	92.50 (47.19–137.81)	0.9560
Lymph, M (Q₁, Q₃)	1.43 (1.02–1.83)	1.46 (1.06–1.85)	1.28 (0.79–1.77)	0.0011
Lymph%, M (Q₁, Q₃)	24.60 (16.99–32.21)	25.00 (17.56–32.44)	20.60 (11.76–29.44)	0.0037
MCH, M (Q₁, Q₃)	29.80 (28.35–31.25)	29.80 (28.40–31.20)	29.35 (27.92–30.78)	0.0012
MCHC, M (Q₁, Q₃)	336.00 (327.50–344.50)	336.00 (328.00–344.00)	331.00 (322.00–340.00)	0.0059
MCV, M (Q₁, Q₃)	88.60 (85.40–91.80)	88.80 (85.66–91.94)	87.55 (83.89–91.21)	0.0066
MONO, M (Q₁, Q₃)	0.52 (0.38–0.66)	0.52 (0.38–0.66)	0.52 (0.39–0.65)	0.8896
MONO%, M (Q₁, Q₃)	8.60 (6.85–10.35)	8.55 (6.80–10.30)	8.95 (7.16–10.74)	0.5577
MPV, M (Q₁, Q₃)	10.20 (9.49–10.91)	10.20 (9.50–10.90)	10.10 (9.31–10.89)	0.0320
ANC, M (Q₁, Q₃)	3.73 (2.60–4.86)	3.73 (2.62–4.84)	3.78 (2.47–5.09)	0.5527
GR%, M (Q₁, Q₃)	62.80 (54.66–70.94)	62.50 (54.76–70.24)	65.45 (55.19–75.71)	0.0067
PCT, M (Q₁, Q₃)	0.23 (0.18–0.28)	0.23 (0.18–0.28)	0.23 (0.20–0.26)	0.4748
PDW, M (Q₁, Q₃)	11.60 (9.89–13.31)	11.70 (10.00–13.40)	11.20 (9.32–13.07)	0.0475
PRL, M (Q₁, Q₃)	222.00 (174.00–270.00)	220.00 (172.00–268.00)	238.00 (194.38–281.62)	0.1099
RBC, M (Q₁, Q₃)	4.25 (3.84–4.66)	4.26 (3.85–4.66)	4.24 (3.84–4.64)	0.1033
RDW-CV, M (Q₁, Q₃)	12.80 (12.05–13.55)	12.80 (12.10–13.50)	13.20 (12.05–14.35)	0.0014
WBC, M (Q₁, Q₃)	6.06 (4.72–7.41)	6.07 (4.74–7.40)	5.87 (4.48–7.26)	0.7074

**Table 3:** Comparison of cytokine-related indicators between PTB and EPTB patients.

Variable	Overall (*n* = 1,580)	TB (*n* = 1,406)	EPTB (*n* = 174)	adj.*p*-value (FDR)
IL-4, M (Q₁, Q₃)	0.01 (0.01, 0.04)	0.01 (0.01, 0.04)	0.01 (0.01, 0.14)	0.6134
IL-6, M (Q₁, Q₃)	3.51 (0.31, 14.85)	3.09 (0.20, 14.32)	5.72 (1.26, 20.67)	0.6134
IL-10, M (Q₁, Q₃)	0.30 (0.01, 1.99)	0.22 (0.01, 1.96)	0.72 (0.01, 2.84)	0.0091
TNF-α, M (Q₁, Q₃)	0.01 (0.01, 0.01)	0.01 (0.01, 0.01)	0.01 (0.01, 0.01)	0.0816
IFN-γ, M (Q₁, Q₃)	0.01 (0.01, 0.13)	0.01 (0.01, 0.12)	0.01 (0.01, 0.59)	0.6134
IL-17a, M (Q₁, Q₃)	0.01 (0.01, 0.01)	0.010(0.01, 0.01)	0.01 (0.01, 0.01)	0.6134
IL-1β, M (Q₁, Q₃)	0.01(0.01, 0.180)	0.01 (0.01, 0.12)	0.01 (0.01, 0.57)	0.6134
IL-5, M (Q₁, Q₃)	0.31 (0.01, 0.72)	0.31 (0.01, 0.72)	0.34 (0.01, 0.78)	0.1425
IFN-α, M (Q₁, Q₃)	0.01(0.01, 0.32)	0.01 (0.01, 0.31)	0.01 (0.01, 0.37)	0.6134
IL-8, M (Q₁, Q₃)	2.89 (0.01, 12.42)	2.80 (0.01, 11.76)	4.12 (0.01, 17.72)	0.6772

**Table 4:** Comparison of lymphocyte subpopulation-related indicator between PTB and EPTB patients.

Variable	Overall (*n* = 1,580)	TB (*n* = 1,406)	EPTB (*n* = 174)	adj.*p*-value (FDR)
CD3+ T cells%, M (Q₁, Q₃)	71.02 (65.11–76.93)	71.13 (65.25–77.02)	70.62 (64.97–76.27)	0.8864
CD3+ T cells, M (Q₁, Q₃)	1,070.00 (730.75–1409.25)	1,082.50 (751.00–1414.00)	885.50 (515.38–1255.62)	0.0008
CD8+/CD45+, M (Q₁, Q₃)	24.00 (18.00–30.00)	24.00 (18.00–30.00)	25.00 (19.12–30.88)	0.7963
CD8+ T, M (Q₁, Q₃)	360.50 (208.75–512.25)	365.00 (213.12–516.88)	294.50 (167.00–422.00)	0.0048
CD4+/CD45+, M (Q₁, Q₃)	41.00 (35.00–47.00)	41.00 (35.00–47.00)	42.00 (35.12–48.88)	0.8864
CD4+ T, M (Q₁, Q₃)	610.00 (403.88–816.12)	622.00 (420.75–823.25)	496.50 (300.62–692.38)	0.0008
CD4+ CD8+/CD45+, M (Q₁, Q₃)	0.20 (0.08–0.32)	0.19 (0.06–0.32)	0.20 (0.07–0.33)	0.8864
CD4+CD8+ T, M (Q₁, Q₃)	3.00 (1.00–5.00)	3.00 (1.00–5.00)	2.00 (0.12–3.88)	0.2081
CD16/56+ NK%, M (Q₁, Q₃)	13.88 (8.10–19.65)	13.77 (8.03–19.51)	15.07 (8.98–21.15)	0.8749
CD16/56+ NK, M (Q₁, Q₃)	199.00 (110.38–287.62)	202.00 (112.00–292.00)	178.00 (85.50–270.50)	0.0723
CD19+ B, M (Q₁, Q₃)	183.00 (93.75–272.25)	185.00 (95.12–274.88)	157.00 (69.88–244.12)	0.0097
CD45+, M (Q₁, Q₃)	1,506.00 (1,073.38–1938.62)	1,529.50 (1,105.50–1953.50)	1,332.00 (822.25–1841.75)	0.0008
Th/Ts (CD4+/CD8+), M (Q₁, Q₃)	1.68 (1.11–2.25)	1.68 (1.11–2.25)	1.63 (1.07–2.19)	0.8864
CD19+ B%, M (Q₁, Q₃)	12.38 (8.33–16.42)	12.32 (8.30–16.33)	12.49 (8.04–16.94)	0.8796

### Feature selection and model performance

**Figure 2:**
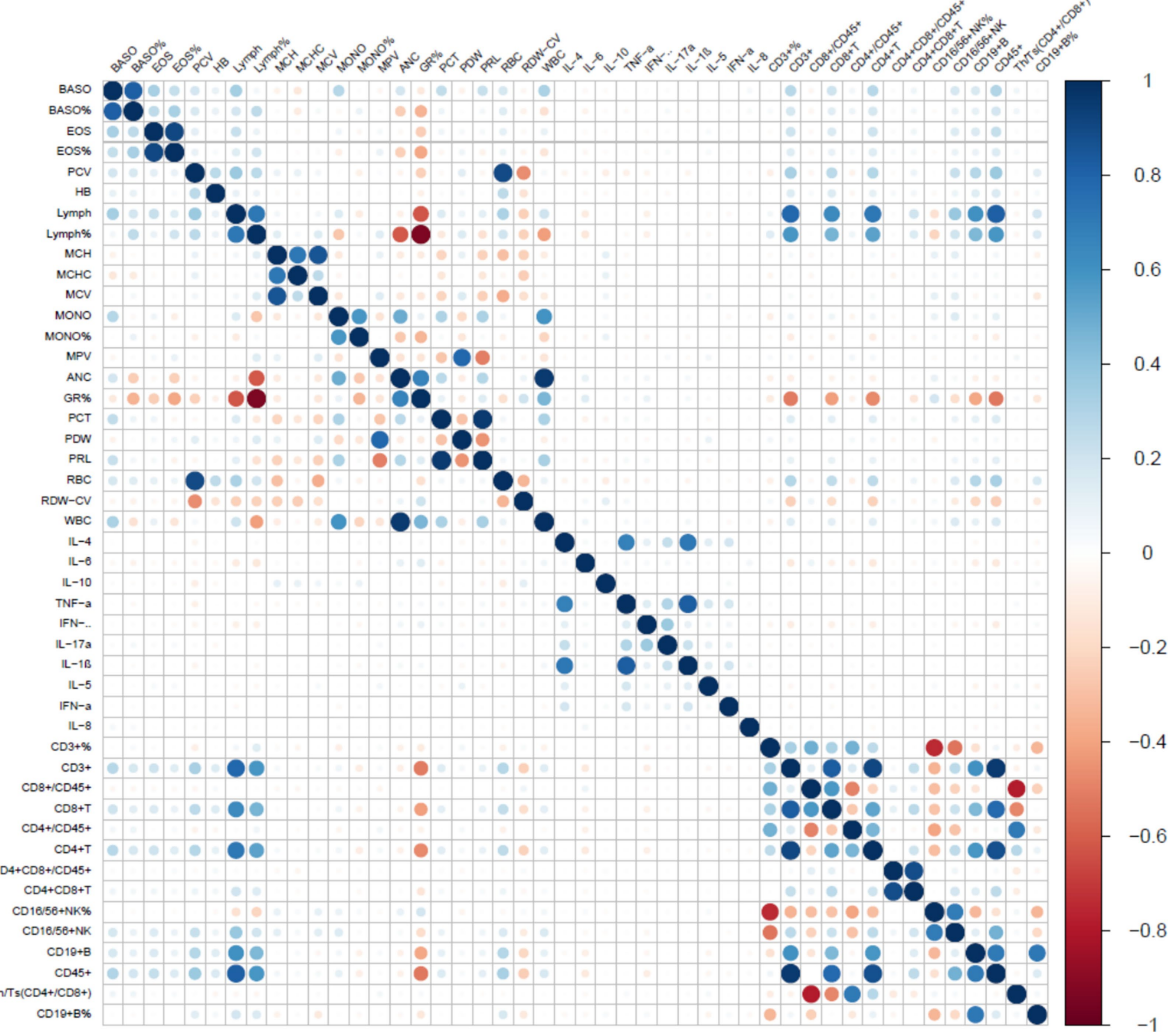
Correlation between characterization factors. The color gradient represents the Pearson correlation coefficient between markers. Blue indicates a positive correlation, red indicates a negative correlation, and the deeper the color (towards dark blue or dark red), the stronger the correlation.

**Figure 3:**
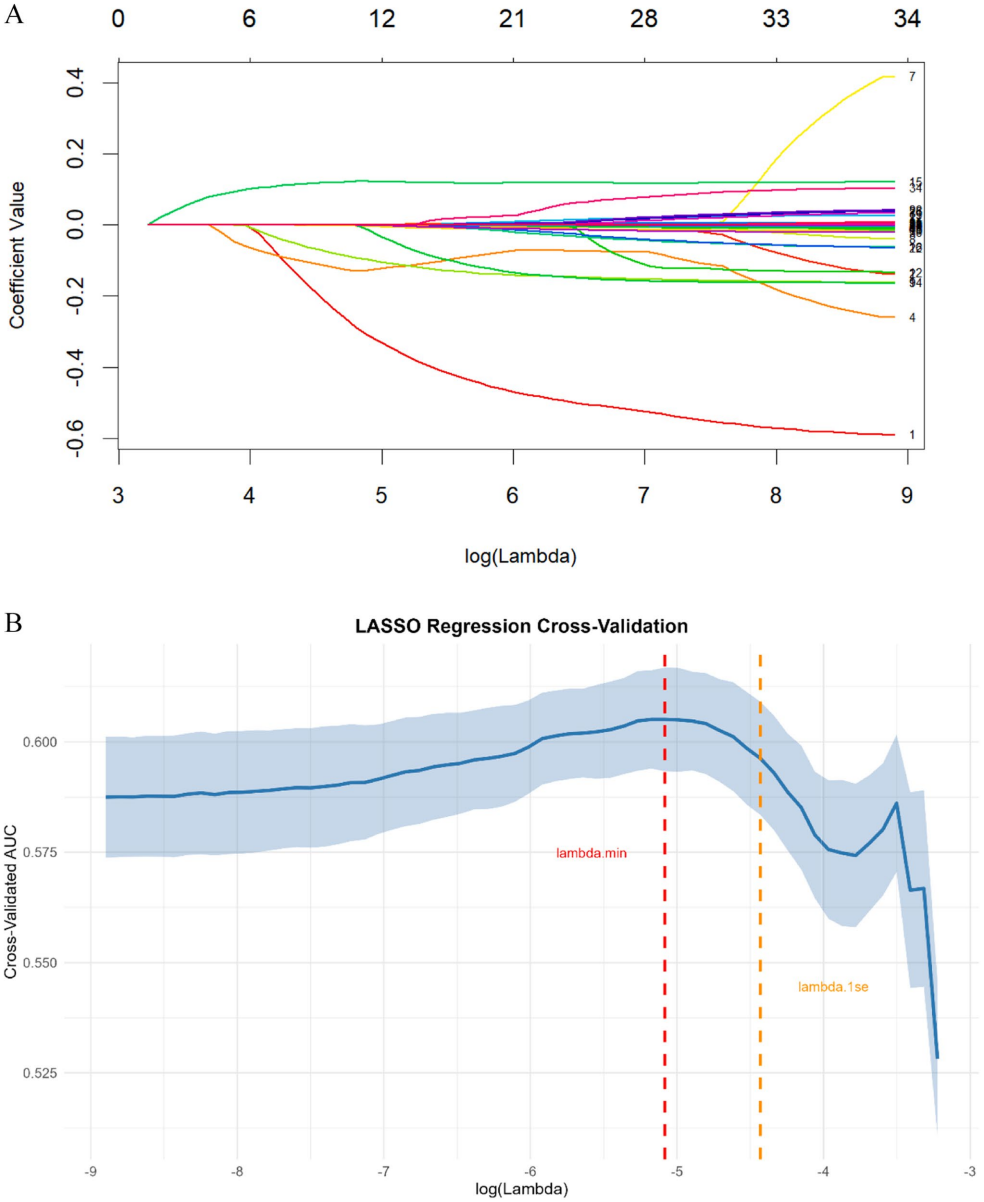
**(A)** LASSO regression coefficient path plot. Each colored line represents a feature, with the x-axis denoting the log-transformed regularization parameter (*λ*) and the *y*-axis representing the coefficient value. **(B)** LASSO regression cross-validation for optimal regularization parameter selection. The solid blue line represents the mean cross-validated AUC, and the shaded area denotes the corresponding standard error. The vertical red dashed line indicates λ.min (the value of λ that minimizes the cross-validation error), while the vertical orange dashed line marks λ.1se (the largest λ where the cross-validated AUC is within one standard error of the minimum error).

After removing constant features, we conducted covariance analysis by calculating Pearson correlation coefficients among candidate features. This analysis revealed substantial multicollinearity among several feature pairs—for example, the correlation coefficient between BASO% and BASO was 0.93, and that between PCV and RBC was 0.88—highlighting the need for further feature selection to address redundancy (Figure 2). Following this, the least absolute shrinkage and selection operator (LASSO) regression algorithm, combined with 5-fold cross-validation, was used to perform a secondary screening of the retained features (Figure 3). The final ML model comprised 12 key features: 8 hematological parameters (BASO, HB, MCHC, MCV, MPV, RBC, Lymph and RDW-CV), 1 cytokine marker (IL-6), and 3 lymphocyte-associated indices (CD4+ T cells, CD8 + T cells and CD4+/CD8+ T-cells).

We trained and evaluated nine machine learning models- Logistic Regression (Log_Reg), Random Forest (RF), Linear Discriminant Analysis (LDA), Support Vector Machine (SVM), Decision Tree (DT), Gradient Boosting Machine (GBM), Naive Bayes, K-Nearest Neighbors (KNN), and XGBoost—on three datasets (Original training set, Balanced training set, Testing set). Performance was assessed using AUC, AUC 95% CI, sensitivity, specificity, PPV, NPV, accuracy, and F1. All metrics are reported for each model and dataset in the table (Table 5; Supplementary Table S1).

**Table 5:** Performance metrics of nine machine learning models across different datasets.

Models	AUC	AUC_CI_lower	AUC_CI_upper	Sensitivity	Specificity	PPV	NPV	Accuracy	F1
Log_Reg	0.828	0.767	0.874	0.712	0.741	0.253	0.954	0.738	0.374
RF	0.764	0.692	0.826	0.635	0.708	0.212	0.94	0.7	0.317
SVM	0.824	0.762	0.875	0.769	0.762	0.286	0.964	0.763	0.417
GBM	0.793	0.717	0.842	0.712	0.722	0.24	0.953	0.721	0.359
XGBoost	0.763	0.682	0.82	0.731	0.675	0.217	0.953	0.681	0.335
NaiveBayes	0.736	0.661	0.803	0.635	0.634	0.176	0.934	0.634	0.276
LDA	0.767	0.696	0.832	0.673	0.694	0.213	0.945	0.691	0.324
DT	0.734	0.638	0.81	0.788	0.789	0.315	0.968	0.789	0.451
KNN	0.846	0.775	0.901	0.769	0.786	0.308	0.965	0.784	0.44

The indexes represented the performance of ML models in the validation set.

Sens, sensitivity; Spec, specificity; PPV, positive predictive value; NPV, negative predictive value; Acc, accuracy; AUC, area under the curve; CI, confidence interval.

**Figure 4:**
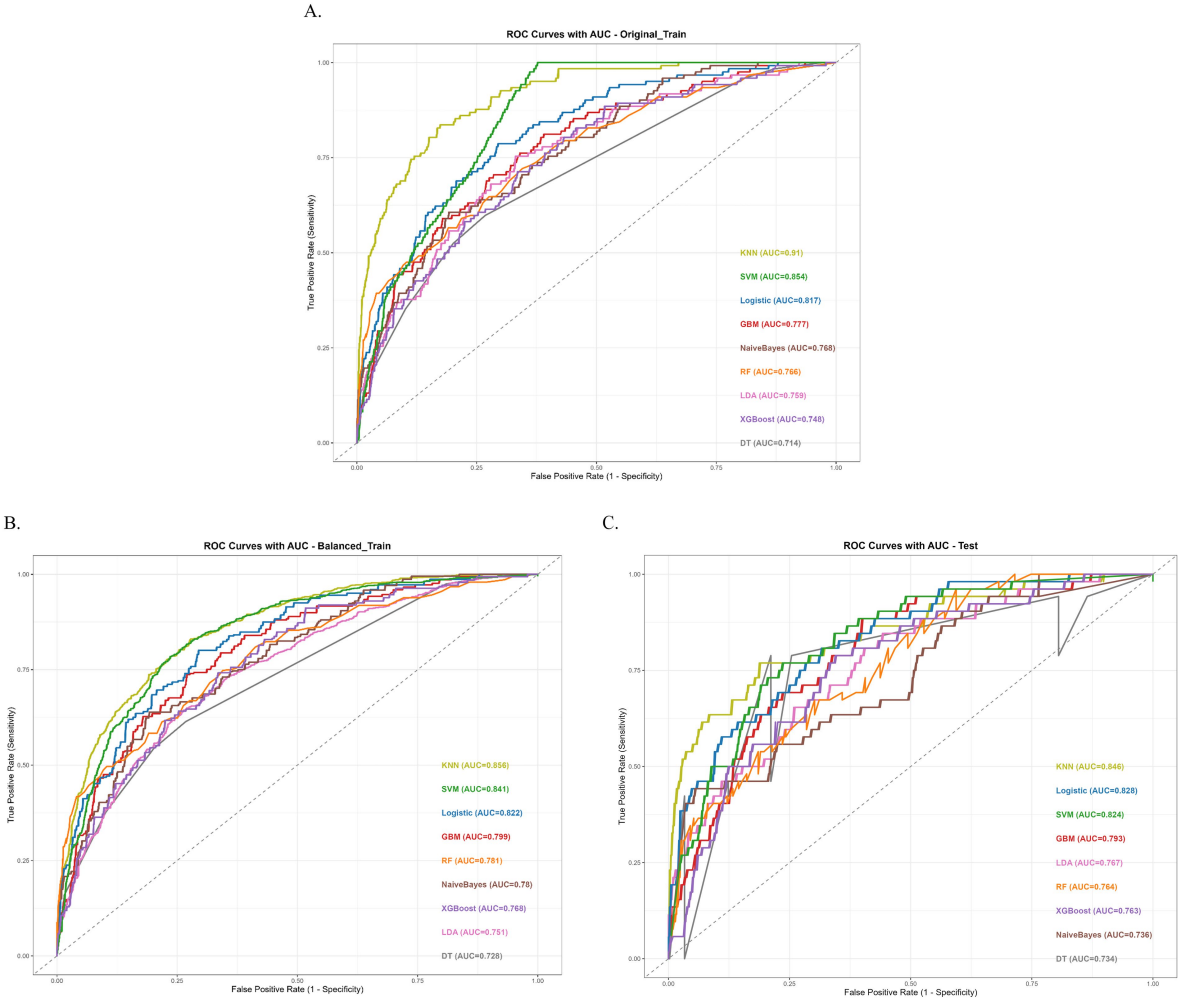
Performance of the nine machine learning models. **(A)** ROC Curves with AUC of original train dataset. **(B)** ROC Curves with AUC of balanced train dataset. **(C)** ROC Curves with AUC of test dataset.

Among the nine machine learning models, the KNN algorithm achieved the best overall performance across the original train (AUC = 0.910, 95% CI: 0.884–0.934), balanced train (AUC = 0.856, 95% CI: 0.839–0.872), and Test (AUC = 0.846, 95% CI: 0.775–0.901) datasets, with balanced sensitivity (0.828/0.768/0.769) and specificity (0.831/0.774/0.786) and the highest F1 scores (0.519/0.770/0.440), indicating strong and generalizable discriminative ability. In the original imbalanced training set, KNN’s nearest-neighbor votes are dominated by the majority class, so most positive samples are surrounded by negatives and easily rank higher than negatives; this inflates the training AUC. After balancing, each class contributes equally to the neighbor pool, forcing KNN to learn a stricter, more generalizable decision boundary; positives are no longer “shielded” by class frequency, so the training AUC decreases (to 0.856) but better reflects cross-sample separability. The SVM model ranked second, with an AUC of 0.854 (95% CI: 0.828–0.880) on the original training set and 0.824 (95% CI: 0.762–0.875) on the test set, but performed unusually high specificity (0.996) and very low sensitivity (0.033). Logistic Regression yielded consistently moderate results (AUC = 0.817 on the original training set; AUC = 0.828 on the test set), while XGBoost (AUC = 0.748/0.763) and Naive Bayes (AUC = 0.768/0.736) showed lower discriminative ability, with most models’ AUCs below 0.80 on the independent test set. Taking into account the performance across all datasets, the KNN model showed high AUC values on the balanced training set, and the test set. Although it may not have been the best in certain metrics (such as PPV), its overall performance was relatively balanced and stable across different datasets. Therefore, the KNN model was selected as the best-performing model for subsequent analysis (Figure 4).

### Model explanation

**Figure 5:**
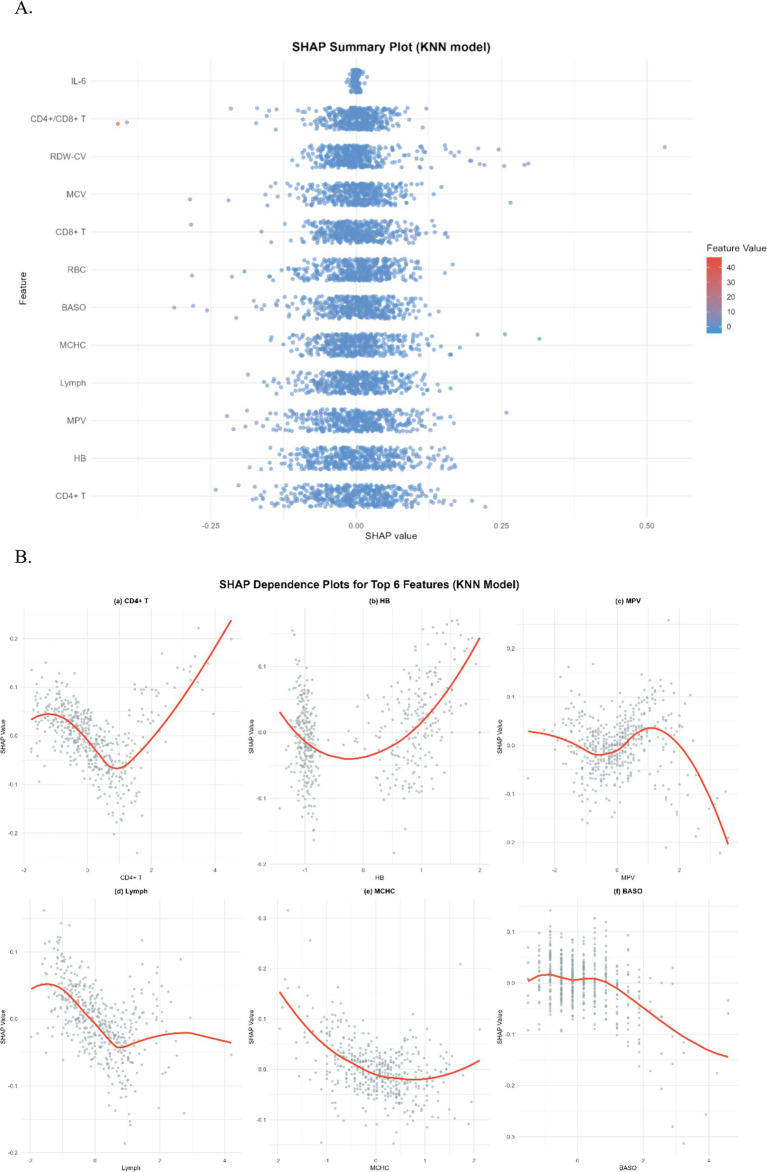
**(A)** SHAP summary plot for KNN model. Each dot represents a single sample’s SHAP value for a feature, with color indicating the feature’s value (blue = low, red = high) and horizontal position indicating the SHAP value’s magnitude. **(B)** SHAP dependence plots for top 6 features in KNN model. Each subplot illustrates the relationship between a feature’s value and its SHAP value, with the red curve representing a LOESS-smoothed trend. Subplots are: (a) CD4+ T cells, (b) Hemoglobin (HB), (c) Mean Platelet Volume (MPV), (d) Lymphocytes (Lymph), (e) Mean Corpuscular Hemoglobin Concentration (MCHC), and (f) Basophils (BASO). These plots elucidate how each feature’s variation contributes to model predictions.

**Figure 6:**
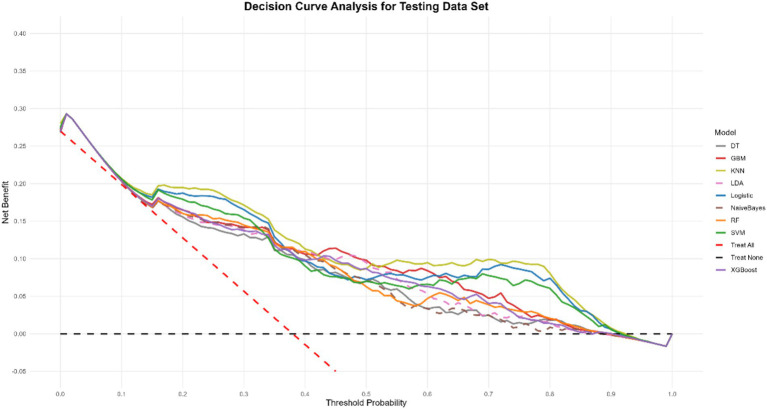
Decision curve analysis of nine models plotting the net benefit at different threshold probabilities.

To gain a more profound understanding and interpretation of the predictive model, we utilize the SHAP analysis to evaluate and visualize the importance of the variables in a KNN model. The SHAP Summary Plot reveals that the top 10 features contributing to the model predictions, ranked by importance, are: CD4+ T cells, HB, MPV, Lymph, MCHC, BASO, RBC, CD8+ T cells, MCV, RDW-CV, CD4+/CD8+ T cells, and IL6 (Figure 5A). Among these, CD4+ T cells emerged as the most influential feature affecting the model’s predictions. Examination of the SHAP dependence plots of the top six important features, it was demonstrated that the relationships between these biomarkers and the model outputs were nonlinear. Specifically, different indicators exhibited varying directional and magnitude effects on the model’s prediction of EPTB or TB across distinct value ranges (Figure 5B). For instance, when CD4+ T cell counts ranged from 0 to 2, the model exhibited a stronger tendency to predict EPTB. This observation suggests that lower CD4+ T cells levels, indicative of relatively impaired immune function, may facilitate the development of EPTB. Conversely, as CD4+ T cells values increased, the corresponding SHAP values rose, indicating that within higher CD4T ranges, the positive influence of CD4+ T cells on model predictions intensified-meaning elevated CD4+ T cells levels also rendered the model more inclined to classify cases as TB. The DCA for the test dataset visually compares the clinical utility of competing models across a range of threshold probabilities (0.0–1.0). Most models (e.g., KNN, Logistic, SVM) demonstrate higher net benefit than the “Treat None” strategy (baseline with zero net benefit) across clinically relevant thresholds (typically 0.1–0.3), indicating their potential value in guiding treatment decisions. Among these, KNN shows the highest net benefit at lower thresholds (~0.1–0.3), closely followed by SVM and Logistic, suggesting they may be preferable for identifying high-risk cases while minimizing unnecessary interventions (Figure 6).

## Discussion

EPTB presents distinct characteristics when compared with PTB. EPTB is marked by atypical clinical manifestations, formidable diagnostic challenges, elevated disability rates, and high mortality. These features not only pose a severe threat to patients’ health but also impose a substantial economic burden on patients, their families, and society at large (18, 19). Whereas the diagnosis of EPTB is recognized as a challenge due to different anatomical locations, uncommon clinical presentations, and insufficient bacterial load in clinical specimens. Resulting in the inadequacy of a single biomarker to differentiate between TB types, a powerful combination of multiple indicators will be the trend for enhanced utility (20).

Leveraging data from the hospital information system on tuberculosis patients, we meticulously organized, analyzed, and ultimately screened 1,580 patients and 11 characteristic factors. Our findings revealed that when the body’s immune system is compromised, *M. tuberculosis* can disseminate through the lymphatic or bloodstream, infiltrating virtually any organ outside the lungs (excluding teeth, hair, and nails), thereby leading to EPTB (21). In response, our selected characterization factors encompass not only conventional blood markers but also immune-related cytokine markers and lymphocyte-related markers. Analysis of the included clinical data further demonstrated a notable reduction in lymphocyte counts among EPTB patients compared to those with PTB. Significant alterations were observed in cytokine-related markers such as IL-6, IL-10, IL-1β, and IL-8, as well as in lymphocyte subpopulation-related markers including CD3+ total T cells, CD8+ T cells, CD4+ T cells, CD16/56+ NK cells, and CD45+ cells.

Previous research has shown that T-lymphocytes play a pivotal role in the immune defense of hosts infected with MTB. Upon MTB infection, T-lymphocytes differentiate into CD4+ and CD8+ T-cells. The initial CD4+ T-cells (Th0) are induced to differentiate into Th1 cells (22, 23). Th1 cells augment cell-mediated anti-infection immunity by secreting various cytokines and cytotoxic T lymphocytes, which in turn activate macrophages to phagocytose MTB within macrophage vesicles (24). For instance, the type 2 cytokine IL-10 is essential for effectively regulating host inflammation (25). Conversely, CD8+ T cells can recognize short peptides and non-peptide antigens, possessing the ability to eliminate intracellular pathogens. MTB-specific CD8+ T cells can exert a cytolytic effect on MTB-infected target cells and induce MTB apoptosis through the secretion of pro-inflammatory cytokines, including perforins, granzymes, and granulysin (26).

A meta-analysis assessing the diagnostic accuracy of the WHO-recommended Xpert Ultra and Xpert MTB/RIF methods for suspected EPTB revealed that these techniques exhibit varying sensitivities depending on the type of extrapulmonary specimen. This highlights their inherent limitations. ML technology, however, offers a potent computational approach capable of handling complex and extensive datasets. ML can process highly variable data and flexibly discern intricate relationships between variables through training (27, 28). In this study, we employed ML techniques to construct nine models, integrating conventional and immune-related testing indicators, to develop models that can accurately discriminate between EPTB and PTB. Through covariance elimination and feature selection, a total of 12 features were ultimately selected. Among the nine ML models, the KNN model demonstrated the most outstanding performance, with an AUC value of 0.846. To further elaborate on the contribution of each feature within the model, we approximated the SHAP values using the Monte Carlo sampling method. The results revealed that CD4+ T cells ranked first among all features, indicating its most significant impact on the model. HB and MPV followed as the second and third, respectively, also exerting substantial influence on the model’s prediction outcomes.

CD4+ T cells are central to adaptive immunity against MTB, particularly in preventing extrapulmonary dissemination. Their protection relies on IFN-γ secretion (activating macrophage bactericidal activity) and coordination of CD8+ T cells, NK cells, and innate immunity to form an “immune barrier” against MTB spread (29). In EPTB, CD4+ T-cell dysfunction—due to early exhaustion, functional failure, or MTB’s immune evasion (e.g., inducing PD-1/CTLA-4 expression, disrupting Th1/Th2 balance to favor Th2 responses/IL-4/IL-10 overproduction, or downregulating MHC-II to impair antigen recognition)—leads to uncontrolled systemic infection (30, 31). CD4+ T cells also maintain granuloma integrity by regulating macrophage polarization, cytokine networks, and Treg activity; their loss in EPTB drives macrophage M2 polarization (reducing bactericidal capacity) and Treg overexpansion, transforming granulomas from protective structures to bacterial sanctuaries (32). HB, while traditionally an oxygen-transport marker, indirectly modulates EPTB immunity/inflammation. Elevated free hemoglobin/iron (from hemolysis/anemia) acts as a pro-oxidant, generating ROS that impair neutrophil/macrophage function and may disrupt antigen presentation, aiding MTB immune evasion. Anemia (common in TB) reduces immune cell metabolism/lymphocyte proliferation, weakening host defenses. HB degradation products further synergize with TLR agonists to trigger excessive TNF-α/IL-6 secretion, causing a dysregulated “inflammatory storm” that disrupts immune cell chemotaxis to extrapulmonary lesions, promotes systemic dysfunction, and facilitates MTB spread (33). At the granuloma level, HB-driven oxidative stress inhibits fibroblast collagen synthesis (weakening structure) and accelerates immune cell apoptosis (collapsing antimicrobial function) (34). MPV, a marker of platelet activation, reflects systemic inflammation in TB. MTB infection stimulates bone marrow to release larger, reactive platelets (raising MPV), which exhibit stronger pro-thrombotic/pro-inflammatory effects via α-granule mediators. In EPTB/tuberculous pleuritis, localized inflammation induces microthrombosis/platelet aggregation, making MPV a dynamic marker of sustained immune activation. Elevated MPV correlates with active disease, higher inflammatory burden, and severe/disseminated TB, linking it to disease progression/complications (35, 36). Our SHAP-based feature importance analysis reveals that: CD4+ T cells are the central regulators of immune control in EPTB, playing well-established roles in IFN-γ-mediated protection, granuloma maintenance, and suppression of immune evasion; HB is a key modulator of immune responses and inflammation, where its dysregulation (either high or low) contributes to immune dysfunction, oxidative damage, and granuloma instability. MPV reflects platelet activation and systemic inflammation, offering a novel perspective on the inflammatory microenvironment in EPTB. These three features not only demonstrate strong predictive power in our model but also align closely with the known biological mechanisms underlying EPTB pathogenesis, immune dissemination, and inflammation.

In summary, our model leverages multidimensional routine laboratory tests to establish a framework for the early diagnosis of EPTB by rapidly integrating available indicators. It has the potential to serve as an adjunct or complementary tool in the diagnosis of tuberculosis.

However, our study has several limitations. First, the data were collected retrospectively from a single center, which may introduce selection bias and limit the generalizability of the findings. Second, we did not have access to an independent or external validation dataset from another hospital or cohort during the study period, and thus the model’s performance in other clinical settings remains to be confirmed. We acknowledge that external validation is essential to establish the robustness and real-world applicability of the model, and this represents a critical direction for future research. Third, despite our efforts to reduce overfitting through feature selection, balancing, and regularization, the retrospective nature of the data poses inherent limitations in causal inference and predictive stability over time. Future prospective, multi-center studies are needed to validate and refine the proposed model.

## Data Availability

The raw data supporting the conclusions of this article will be made available by the authors, without undue reservation.

## Supplementary material

SUPPLEMENTARY TABLE S1 Performance metrics of nine machine learning models on the original training set, balanced training set, and test set of different datasets.

## References

[1] World Health Organization. Global tuberculosis report 2024. Geneva: WHO (2024).

[2] Churchyard G Kim P Shah NS Rustomjee R Gandhi N Mathema B . What we know about tuberculosis transmission: An overview. J Infect Dis. (2017) 216:S629–35. doi: 10.1093/infdis/jix362, 29112747 PMC5791742

[3] Stewart GR Robertson BD Young DB. Tuberculosis: a problem with persistence. Nat Rev Microbiol. (2003) 1:97–105. doi: 10.1038/nrmicro749, 15035039

[4] Jones BE Young SM Antoniskis D Davidson PT Kramer F Barnes PF. Relationship of the manifestations of tuberculosis to CD4 cell counts in patients with human immunodeficiency virus infection. Am Rev Respir Dis. (1993) 148:1292–7. doi: 10.1164/ajrccm/148.5.1292, 7902049

[5] Lewinsohn DA Gennaro ML Scholvinck L Lewinsohn DM. Tuberculosis immunology in children: diagnostic and therapeutic challenges and opportunities. Int J Tuberc Lung Dis. (2004) 8:658–74. Available online at: https://www.ingentaconnect.com/content/iuatld/ijtld/2004/00000008/00000005/art00025;jsessionid=4w0efk4cbs9md.x-ic-live-01 15137550

[6] Yang Q Han J Shen J Peng X Zhou L Yin X. Diagnosis and treatment of tuberculosis in adults with HIV. Medicine. (2022) 101:e30405. doi: 10.1097/md.0000000000030405, 36107594 PMC9439776

[7] Esmail H Riou C Bruyn ED du Bruyn E Lai RP-J Harley YXR . The immune response to *Mycobacterium tuberculosis* in HIV-1-coinfected persons. Annu Rev Immunol. (2018) 36:603–38. doi: 10.1146/annurev-immunol-042617-053420, 29490165

[8] Baykan AH Sayiner HS Aydin E Koc M Inan I Erturk SM. Extrapulmonary tuberculosıs: an old but resurgent problem. Insights Imaging. (2022) 13:39. doi: 10.1186/s13244-022-01172-0, 35254534 PMC8901940

[9] Heye T Stoijkovic M Kauczor HU Junghanss T Hosch W. Extrapulmonary tuberculosis: radiological imaging of an almost forgotten transformation artist. Rofo. (2011) 183:1019–29. doi: 10.1055/s-0031-1273429, 21667424

[10] Steingart KR Henry M Laal S Hopewell PC Ramsay A Menzies D . A systematic review of commercial serological antibody detection tests for the diagnosis of extrapulmonary tuberculosis. Thorax. (2007) 83:705–12. doi: 10.1136/thx.2006.075754, 17989270 PMC2734443

[11] Pai M Nicol MP Boehme CC. Tuberculosis diagnostics: state of the art and future directions. Microbiol Spectr. (2016) 4:10.1128/microbiolspec.tbtb2-0019-2016. doi: 10.1128/microbiolspec.TBTB2-0019-2016, 27763258

[12] Bera K Schalper KA Rimm DL Velcheti V Madabhushi A. Artificial intelligence in digital pathology - new tools for diagnosis and precision oncology. Nat Rev Clin Oncol. (2019) 16:703–15. doi: 10.1038/s41571-019-0252-y, 31399699 PMC6880861

[13] Jordan MI Mitchell TM. Machine learning: trends, perspectives, and prospects. Science. (2015) 349:255–60. doi: 10.1126/science.aaa8415, 26185243

[14] Luo Y Xue Y Liu W Song H Huang Y Tang G . Development of diagnostic algorithm using machine learning for distinguishing between active tuberculosis and latent tuberculosis infection. BMC Infect Dis. (2022) 22:965. doi: 10.1186/s12879-022-07954-7, 36581808 PMC9798640

[15] Shao J Ma J Yu Y Zhang S Wang W Li W . A multimodal integration pipeline for accurate diagnosis, pathogen identification, and prognosis prediction of pulmonary infections. Innovation. (2024) 5:100648. doi: 10.1016/j.xinn.2024.100648, 39021525 PMC11253137

[16] Zhou X Lu P Zheng Z Tolliver D Keramati A. Accident prediction accuracy assessment for highway-rail grade crossings using random forest algorithm compared with decision tree. Reliab Eng Syst Saf. (2020) 200:106931. doi: 10.1016/j.ress.2020.106931

[17] Mullick SS Datta S Das S. Adaptive learning-based -nearest neighbor classifiers with resilience to class imbalance. IEEE Trans Neural Netw Learn Syst. (2018) 29:5713–25. doi: 10.1109/tnnls.2018.2812279, 29993560

[18] Banta JE Ani C Bvute KM Lloren JIC Darnell TA. Pulmonary vs. extra-pulmonary tuberculosis hospitalizations in the US [1998–2014]. J Infect Public Health. (2020) 13:131–9. doi: 10.1016/j.jiph.2019.07.001, 31422038

[19] Prasanna T Kathiresan J Palanivel C Yogesh B Kavita V Das M. Catastrophic costs of tuberculosis care: a mixed methods study from Puducherry, India. Glob Health Action. (2018) 11:1477493. doi: 10.1080/16549716.2018.1477493, 29902134 PMC6008578

[20] Dahiya B Mehta N Soni A Mehta PK. Diagnosis of extrapulmonary tuberculosis by GeneXpert MTB/RIF ultra assay. Expert Rev Mol Diagn. (2023) 23:561–82. doi: 10.1080/14737159.2023.2223980, 37318829

[21] Li T Yan X Du X Huang F Wang N Ni N . Extrapulmonary tuberculosis in China: a national survey. Int J Infect Dis. (2023) 128:69–77. doi: 10.1016/j.ijid.2022.12.005, 36509333

[22] Singh A Dey AB Mohan A Mitra DK. Programmed death-1 receptor suppresses γ-IFN producing NKT cells in human tuberculosis. Tuberculosis (Edinb). (2014) 94:197–206. doi: 10.1016/j.tube.2014.01.005, 24629634

[23] Coulter F Parrish A Manning D Kampmann B Mendy J Garand M . IL-17 production from T helper 17, mucosal-associated invariant T, and γδ cells in tuberculosis infection and disease. Front Immunol. (2017) 8:1252. doi: 10.3389/fimmu.2017.01252, 29075255 PMC5641565

[24] Boom WH Schaible UE Achkar JM. The knowns and unknowns of latent *Mycobacterium tuberculosis* infection. J Clin Invest. (2021) 131:e136222. doi: 10.1172/JCI136222, 33529162 PMC7843221

[25] Murray PJ Young RA. Increased antimycobacterial immunity in interleukin-10-deficient mice. Infect Immun. (1999) 67:3087–95. doi: 10.1128/iai.67.6.3087-3095.1999, 10338525 PMC96626

[26] Patankar YR Sutiwisesak R Boyce S Lai R Lindestam Arlehamn CS Sette A . Limited recognition of *Mycobacterium tuberculosis*−infected macrophages by polyclonal CD4 and CD8 T cells from the lungs of infected mice. Mucosal Immunol. (2020) 13:140–8. doi: 10.1038/s41385-019-0217-6, 31636345 PMC7161428

[27] Xing Z Ding W Zhang S Zhong L Wang L Wang J . Machine learning-based differentiation of nontuberculous mycobacteria lung disease and pulmonary tuberculosis using CT images. Biomed Res Int. (2020) 2020:6287545. doi: 10.1155/2020/6287545, 33062689 PMC7545409

[28] Zhou Z Zhou X Cheng L Wen L An T Gao H . Machine learning algorithms utilizing blood parameters enable early detection of immunethrombotic dysregulation in COVID-19. Clin Transl Med. (2021) 11:e523. doi: 10.1002/ctm2.523, 34586734 PMC8473644

[29] Silveira-Mattos PS Barreto-Duarte B Vasconcelos B Fukutani KF Vinhaes CL Oliveira-De-Souza D . Differential expression of activation markers by *Mycobacterium tuberculosis*-specific CD4+ T cell distinguishes extrapulmonary from pulmonary tuberculosis and latent infection. Clin Infect Dis. (2020) 71:1905–11. doi: 10.1093/cid/ciz1070, 31665254 PMC8463092

[30] Jasenosky LD Scriba TJ Hanekom WA Goldfeld AE. T cells and adaptive immunity to *Mycobacterium tuberculosis* in humans. Immunol Rev. (2015) 264:74–87. doi: 10.1111/imr.12274, 25703553

[31] Becker SH Ronayne CE Bold TD Jenkins MK. CD4 (+) T cells recruit, then engage macrophages in cognate interactions to clear *Mycobacterium tuberculosis* from the lungs. bioRxiv. (2024). doi: 10.1101/2024.08.22.609198, 39229103 PMC11370583

[32] Bold TD Banaei N Wolf AJ Ernst JD. Suboptimal activation of antigen-specific CD4+ effector cells enables persistence of *M. tuberculosis* in vivo. PLoS Pathog. (2011) 7:e1002063. doi: 10.1371/journal.ppat.1002063, 21637811 PMC3102708

[33] Nathan C Ding A. Nonresolving inflammation. Cell. (2010) 140:871–82. doi: 10.1016/j.cell.2010.02.029, 20303877

[34] Drakesmith H Prentice A. Viral infection and iron metabolism. Nat Rev Microbiol. (2008) 6:541–52. doi: 10.1038/nrmicro1930, 18552864

[35] Gunluoglu G Yazar EE Veske NS Seyhan EC Altin S. Mean platelet volume as an inflammation marker in active pulmonary tuberculosis. Multidiscip Respir Med. (2014) 9:11. doi: 10.1186/2049-6958-9-11, 24581084 PMC3995664

[36] Sonmez O Sonmez M. Role of platelets in immune system and inflammation. Porto Biomed J. (2017) 2:311–4. doi: 10.1016/j.pbj.2017.05.005, 32258788 PMC6806752

